# Tup1 Paralog *CgTUP11* Is a Stronger Repressor of Transcription than *CgTUP1* in Candida glabrata

**DOI:** 10.1128/msphere.00765-21

**Published:** 2022-03-28

**Authors:** Lilian N. Bui, Christine L. Iosue, Dennis D. Wykoff

**Affiliations:** a Department of Biology, Villanova University, Villanova, Pennsylvania, USA; University of Georgia

**Keywords:** candidiasis, transcription factors, sorbitol, whole-genome duplication, chromatin, candidiasis, transcription factor, whole-genome duplication

## Abstract

*TUP1* is a well-characterized repressor of transcription in Saccharomyces cerevisiae and Candida albicans and is observed as a single-copy gene. We observe that most species that experienced a whole-genome duplication outside of the *Saccharomyces* genus have two copies of *TUP1* in the *Saccharomycotina* yeast clade. We focused on Candida glabrata and demonstrated that the uncharacterized *TUP1* homolog, C. glabrata *TUP11* (*CgTUP11*), is most like the S. cerevisiae *TUP1* (*ScTUP1*) gene through phenotypic assays and transcriptome sequencing (RNA-seq). Whereas *CgTUP1* plays a role in gene repression, it is much less repressive in standard growth media. Through RNA-seq and reverse transcription-quantitative PCR (RT-qPCR), we observed that genes associated with pathogenicity (*YPS2*, *YPS4*, and *HBN1*) are upregulated upon deletion of either paralog, and loss of both paralogs is synergistic. Loss of the corepressor *CgCYC8* mimics the loss of both paralogs, but not to the same extent as the *Cgtup1*Δ *Cgtup11*Δ mutant for these pathogenesis-related genes. In contrast, genes involved in energy metabolism (*CgHXT2*, *CgADY2*, and *CgFBP1*) exhibit similar behavior (dependence on both paralogs), but deletion of *CgCYC8* is very similar to the *Cgtup1*Δ *Cgtup11*Δ mutant. Finally, some genes (*CgMFG1* and *CgRIE1*) appear to only be dependent on *CgTUP11* and *CgCYC8* and not *CgTUP1*. These data indicate separable and overlapping roles for the two *TUP1* paralogs and that other genes may function as the *Cg*Cyc8 corepressor. Through a comparison by RNA-seq of *Sctup1*Δ, it was found that *TUP1* homologs regulate similar genes in the two species. This work highlights that studies focused only on *Saccharomyces* may miss important biological processes because of paralog loss after genome duplication.

**IMPORTANCE** Due to a whole-genome duplication, many yeast species related to C. glabrata have two copies of the well-characterized *TUP1* gene, unlike most *Saccharomyces* species. This work identifies roles for the paralogs in C. glabrata, highlights the importance of the uncharacterized paralog, called *TUP11*, and suggests that the two paralogs have both overlapping and unique functions. The *TUP1* paralogs likely influence pathogenicity based on *tup* mutants upregulating genes that are associated with pathogenicity.

## INTRODUCTION

Candida glabrata and Saccharomyces cerevisiae are closely related species in the Ascomycota phylum with few, but significant, differences in environment and metabolism (1). C. glabrata is an opportunistic pathogen, has differences in drug and stress resistance and adherence relative to S. cerevisiae, and it is the second leading cause of candidiasis in the United States (2–4). The common ancestor of C. glabrata and S. cerevisiae underwent a whole-genome duplication (WGD) event, with both species losing most of these paralogs (often called ohnologs) (5–7). S. cerevisiae appears to have lost many transcription factor duplicates in particular, leading to the hypothesis that S. cerevisiae might have a simpler transcriptional network relative to other related yeast species (8). Preservation of the two paralogs suggests function and raises the following questions: what duplicates are maintained, and do those duplicates have an impact on growth characteristics?

Tup1 has been well characterized as a global transcription repressor in S. cerevisiae. Mutants that were able to take up dTMP were designated *tup*, for thymidine uptake mutants (9). Additionally, *tup1* mutants were identified while screening for genes that regulate the mating-type locus (10). Tup1 belongs to a family of WD repeat repressor proteins (11, 12). On the C-terminal end, there are seven repeats of 43 amino acids with highly conserved residues, which are believed to be essential to Tup1 function (13). In contrast, the N-terminal is not as critical for repression (14). In S. cerevisiae, one unit of Tup1 works with four units of Cyc8 (also known as Ssn6) in the Tup1-Cyc8 repressor system (15).

The mechanism of Tup1-Cyc8 repression in S. cerevisiae is well studied. The complex represses over 150 yeast genes and up to 3% of S. cerevisiae genes, including diverse genes related to glucose metabolism, oxygen availability, and DNA damage (16–18). Tup1-Cyc8 affects transcription broadly through several proposed mechanisms. It is thought that sequence-specific DNA binding proteins recruit the system to promoters, but Cyc8 and Tup1 have different roles in repression. Cyc8 generally interacts more directly with the binding proteins whereas Tup1 facilitates other protein interactions, leading to repression of transcription (16, 19). While evidence suggests that the complex inhibits RNA polymerase II function, it also prevents transcription through epigenetic mechanisms. The Tup1-Cyc8 repressor system interacts with multiple class I histone deacetylases, making DNA less accessible for transcription (20). More specifically, Tup1 interacts with histones H3 and H4 to remodel chromatin (21, 22). Thus, while there are likely multiple mechanisms by with the Tup1-Cyc8 complex represses transcription, the complex is important for repressing important genes to the cell.

When either *TUP1* or *CYC8* is deleted in S. cerevisiae, mutants exhibit phenotypes related to the inappropriate expression of genes (18). For example, S. cerevisiae *cyc8* and *tup1* mutants flocculate, are defective in sporulation (13), and exhibit temperature-dependent phenotypes (23). Phenotypes are also noted in the presence of different carbon sources. For example, *Sctup1* mutants can efficiently assimilate sorbitol, unlike their wild-type counterparts (24), and Tup1 is implicated in maltose metabolism (25).

C. glabrata has maintained two copies of *TUP1* (*CAGL0C03608g* and *CAGL0E00561g*), whereas S. cerevisiae has lost one. Studies with *TUP1* in C. glabrata have only focused on *CAGL0C03608g* (26); however, an uncharacterized paralog exists (we name this paralog *CAGL0E00561g* [*CgTUP11*]), raising the question of why these two paralogs have been preserved over evolutionary time. A phenotype has not been associated with the *CgTUP1* or the *CgTUP11* gene in C. glabrata, and the aim of this project was to explore their functions by looking for a phenotype for C. glabrata *TUP1* and *TUP11* mutants and determining what genes are regulated by the paralogs using transcriptome sequencing (RNA-seq). Additionally, we aimed to uncover which paralog is most similar in function to *ScTUP1* and how loss of *TUP1* homologs in each species impacts gene expression.

## RESULTS

### Many yeast species that experienced a whole-genome duplication have retained two copies of *TUP1*.

**FIG 1:**
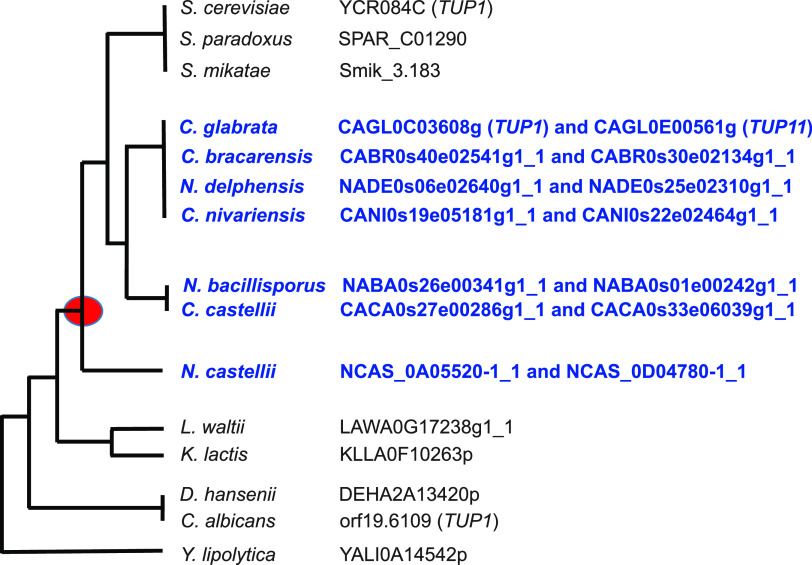
Characterization of Tup1 homologs in the *Saccharomycotina* clade. Using a simplified phylogenetic tree (32, 33), where the red circle represents the whole-genome duplication, we performed BLASTp on the genomes of the 16 species identified by (34). With visual inspection of potential homologs, we determined that >50% identity over ∼400 aa was a suitable cutoff to identify *TUP1* orthologs. The species names in blue indicate species where there are two copies of *TUP1* in the genome. Beside the names of the species are the systematic names of the identified genes.

Examination of the C. glabrata genome indicated two paralogs related to *ScTUP1*. The two paralogs are CAGL0C03608g (643 amino acids [aa]), also annotated as *CgTUP1*, which has 69% identity with *ScTUP1* (713 aa), and CAGL0E00561g (836 aa), which has 67% identity with *ScTUP1*. Of note, both paralogs share significant similarity over regions corresponding to the N and C terminals. Whereas many proteins share some similarity with the WD domain repeats in the C terminus (14), clear Tup1 homologs exhibit at least 50% identity over at least 400 aa of alignment. To determine which yeast species have more than one copy of *TUP1* in the genome, we used *ScTUP1*, *CgTUP1*, and *CgTUP11* to BLASTp search various pre-WGD and post-WGD genomes in the *Saccharomycotina* clade (Fig. 1). In the pre-WGD species, no species appear to have more than one copy of a *TUP1* homolog, and in the post-WGD species, there is a clear division. The *Saccharomyces* species have one copy, and the other post-WGD species have two copies. While only correlative, these results suggest that there is a selective advantage to having two copies of *TUP1* in the post-WGD species.

### Deletion of the two C. glabrata *TUP1* homologs results in few clear phenotypes, but *TUP11* appears to functionally replace *ScTUP1*.

To begin to understand the advantage of having two copies of *TUP1*, we deleted both paralogs, singly and in combination, as well as *CYC8* in C. glabrata. We have named *CAGL0E00561g TUP11* to indicate its relation to *CgTUP1* (*CAGL0C03608g*). Characterization of multiple deletion strains indicated that unlike for *Sctup1*Δ strains, which rapidly flocculate and precipitate to the bottom of a culture tube, there is no clear flocculation phenotype. However, the growth rate of the double deletion strain is lower than for either single mutant or the wild type (see Fig. S1A in the supplemental material). Thus, we began an extensive screen for potential phenotypes.

10.1128/mSphere.00765-21.1 FIG S1 Growth of S. cerevisiae and C. glabrata strains in synthetic defined (SD) medium with 2% glucose (A), 0.2% glucose (B), 2% ethanol replacing glucose (C), or 1.5 M potassium chloride (KCl) (D) added to standard medium. Logarithmically growing cells in SD with 2% glucose were washed with water, inoculated under various growth conditions at an optical density at 600 nm (OD_600_) of 0.01, and grown at 30°C in 96-well plates. OD_600_ was measured after 24 h for 2% glucose and after 48 h for the remaining conditions. The data presented are the means and standard deviations of six biological replicates. *P* value was determined by a Student *t* test comparing each species’ mutants to the wild-type strain, with a single asterisk indicating a *P* value of <0.05 and a double asterisk indicating a *P* value of <0.01. Download FIG S1, EPS file, 1.7 MB. Copyright © 2022 Bui et al. 2022 Bui et al. https://creativecommons.org/licenses/by/4.0/ This content is distributed under the terms of the Creative Commons Attribution 4.0 International license.

Streaking strains on agar plates, we were unable to determine clear phenotypes for the *Cgtup1*Δ, *Cgtup11*Δ, *Cgtup1*Δ *tup11*Δ, and *Cgcyc8*Δ mutants under the following conditions: temperature sensitivity, growth in added salts such as CaCl_2_, FeCl_3_, or KCl, growth in altered pH (pH = 2 or pH = 7), and sensitivity to ketoconazole and 2-deoxyglucose. The mutants behaved like the wild type, with only subtle growth defects in some conditions. We then chose a few conditions where there might have been a subtle phenotype on plates, grew the strains in liquid medium, and quantified growth (Fig. S1). We present a few quantified examples to demonstrate the variability in growth assays under different conditions. Of note is the statistically significant difference between the wild type and the *Cgtup1*Δ *tup11*Δ mutant, which does have a phenotype similar to that of the *Sctup1*Δ strain under standard growth conditions (synthetic medium with 2% glucose) and in 0.2% glucose. Additionally, we observed a growth enhancement in the *Cgtup1*Δ and *Cgcyc8*Δ mutants relative to the wild type in 2% ethanol; however, all cells grow poorly under this growth condition. Through multiple biological replicates we observed a lot of variability, and so while there was statistical significance in some mutants in some conditions, we do not feel comfortable asserting that there is a strong phenotype. We conclude that there are differences between wild-type and C. glabrata mutants but few phenotypes are easily observable.

**FIG 2:**
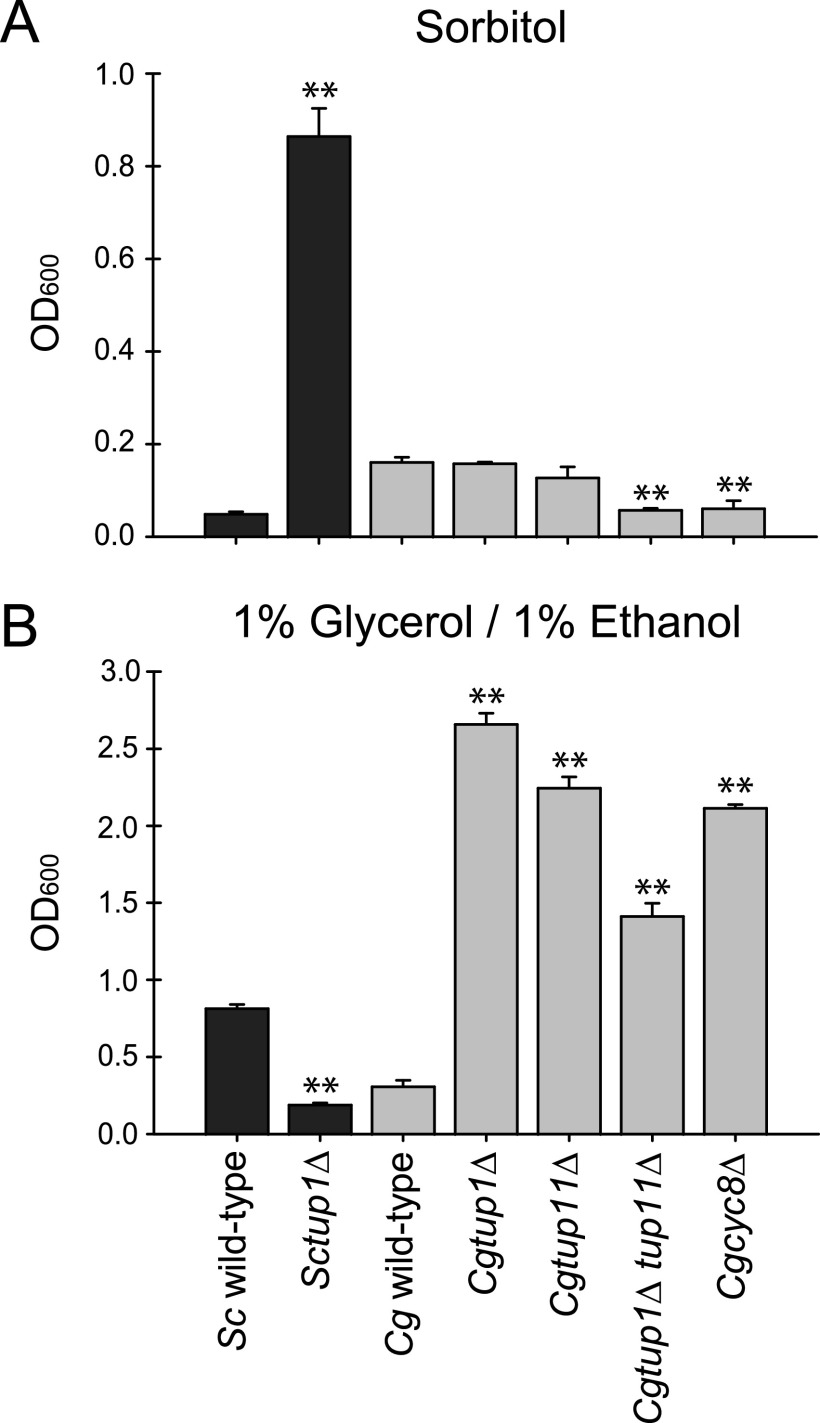
Growth of S. cerevisiae and C. glabrata strains in medium where sorbitol replaces glucose (A) or 1% glycerol/1% ethanol replaces glucose (B). (A) Cells of each strain were pregrown overnight at 30°C in liquid YEP with 3% glycerol to logarithmic growth phase, then washed, and inoculated into synthetic defined medium (SD) with 2% sorbitol replacing glucose at an optical density at 600 nm (OD_600_) of 0.05. OD_600_ was measured after 48 h. We were unable to observe the same phenotypes in YEP plus 2% sorbitol, indicating that medium composition is crucial to the observed benefit of sorbitol to the *Sctup1*Δ mutant. (B) Logarithmically growing cells in SD with 2% glucose were washed and inoculated into SD with 1% glycerol/1% ethanol replacing glucose at an OD_600_ of 0.05 and grown. OD_600_ was measured after 24 h. For both panels A and B, the data presented are the means and standard deviations of three biological replicates. *P* value was determined by a Student *t* test comparing each species’ mutants to the wild-type strain, with a single asterisk indicating a *P* value of <0.05 and a double asterisk indicating a *P* value of <0.01. Standard growth of the strains was confirmed in SD medium with 2% glucose (data not shown).

To confirm that we were incubating cells under conditions that could uncover a phenotype, we focused on growth in added sorbitol (24). We grew mutants and the wild type under conditions where sorbitol replaced glucose and confirmed that the *Sctup1*Δ mutant was able to grow much better than the wild type (Fig. 2A). Phenotypes where the mutant grows better are more convincing because the mutants often are somewhat sicker than the wild type under standard growth conditions (Fig. S1A). We then compared the C. glabrata strains and noted that none were able to grow better than the wild type in the same medium; however, the C. glabrata double mutant and the *Cgcyc8*Δ mutant do have a statistically significant defect in growth relative to single mutants or the wild type. We conclude that deletion of the *CgTUP1* paralogs or the *CgCYC8* gene does not confer the ability to grow in sorbitol in C. glabrata, unlike in S. cerevisiae. During our screening for phenotypes, we did uncover a phenotype for C. glabrata mutants in the presence of nonfermentable carbon sources. In the presence of 2% ethanol, the *Cgtup1*Δ mutant and *Cgcyc8*Δ mutant grew better than the wild type or other mutants, but because growth was highly retarded, we do not feel comfortable concluding that it is a strong phenotype (Fig. S1C). However, when we grew C. glabrata in 1% glycerol/1% ethanol, we observed a robust phenotype when (i) either *TUP* paralog was deleted, (ii) both were deleted, or (iii) *CgCYC8* was deleted (Fig. 2B). We conclude that mutants of both species are capable of having advantages in alternative carbon sources, just different ones.

**FIG 3:**
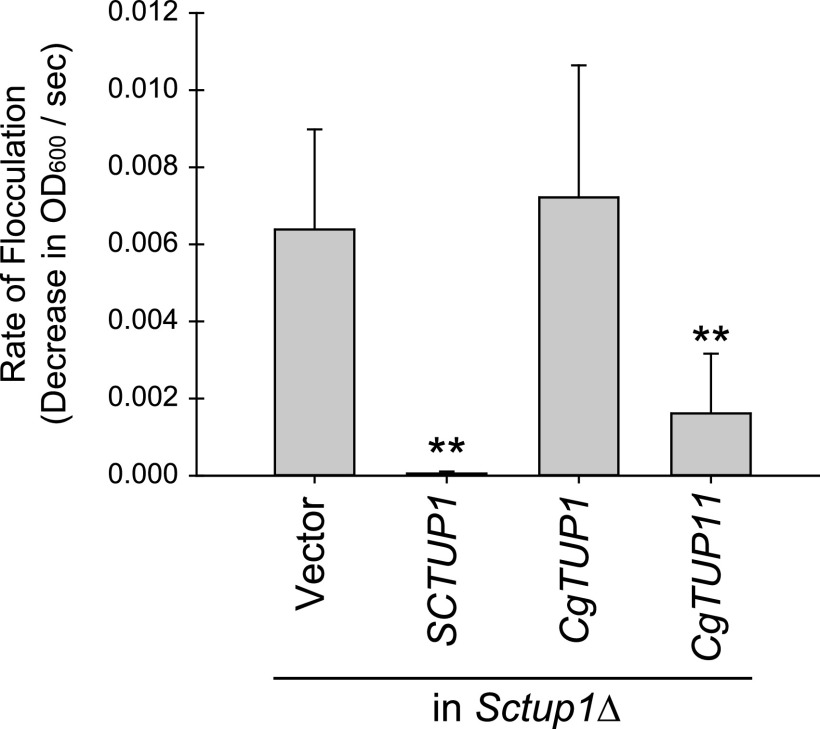
*CgTUP11* is capable of suppressing the flocculation phenotype of the *Sctup1*Δ strain. The change in OD_600_ over time was used to quantify flocculation rates. The *Sctup1*Δ strain was transformed with either plasmid alone (a *URA3*^+^ vector) or plasmids containing *ScTUP1*, *CgTUP1*, or *CgTUP11* and grown in liquid synthetic defined (SD) growth medium without uracil for ∼20 h at 30°C. Each sample was vortexed for 30 s, then the OD_600_ was read every 15 s for 1 min, and the slope of the decline was measured. The means and standard deviations of six biological replicates are presented. *P* value was determined by a Student *t* test comparing each *TUP*-containing plasmid to the vector alone strain, with a single asterisk indicating a *P* value of <0.05 and a double asterisk indicating a *P* value of <0.01.

We next assessed whether the C. glabrata genes were capable of complementing an *Sctup1*Δ strain. A clear phenotype observable with *Sctup1*Δ mutants is the rapid settling of a culture in liquid medium because of increased flocculation. To determine which C. glabrata paralog would complement the flocculation phenotype, we measured spectrophotometrically the loss of absorbance from a culture as the cells settled by measuring the optical density at 600 nm (OD_600_) every 15 s. We then derived slopes of loss of absorbance to quantify the flocculation phenotype (Fig. 3). We cloned *ScTUP1*, *CgTUP1*, and *CgTUP11* into a plasmid and transformed each plasmid into an *Sctup1*Δ strain. We determined that the *ScTUP1* plasmid suppressed the flocculation phenotype, as expected, and *CgTUP11* largely suppressed the flocculation, while *CgTUP1* did not. We confirmed that the cloned genes are functional by looking at complementation in C. glabrata *tup* mutant strains when grown in medium where 1% glycerol/1% ethanol replaced glucose (Fig. S2). As shown in Fig. 2B, the *Cgtup1Δ* and *Cgtup11Δ* strains grew better than the wild type in the presence of 1% glycerol/1% ethanol. Adding back *CgTUP1* on a plasmid to a *Cgtup1Δ* mutant or *CgTUP11* on a plasmid to a *Cgtup11Δ* mutant restored growth to wild-type levels.

10.1128/mSphere.00765-21.2 FIG S2 *CgTUP1* and *CgTUP11* complement the *Cgtup1Δ* and *Cgtup11Δ* strains, respectively, in medium where 1% glycerol/1% ethanol replaces glucose. C. glabrata *tup* mutants were transformed with either plasmid alone (a *HIS3^+^* vector) or a plasmid containing *CgTUP1* (in *Cgtup1*Δ) or *CgTUP11* (in *Cgtup11*Δ). Logarithmically growing cells in SD without histidine with 2% glucose were washed and inoculated into SD with 1% glycerol/1% ethanol replacing glucose at an OD_600_ of 0.05 and grown at 30°C. OD_600_ was measured after 24 h. The data presented are the means and standard deviations of three biological replicates. *P* value was determined by a Student *t* test comparing each deletion strain with plasmid to the wild-type strain, with a single asterisk indicating a *P* value of <0.05 and a double asterisk indicating a *P* value of <0.01. It should be noted that while the *Cgtup1Δ* strain with vector does not have an asterisk indicating significance, the *P* value for this strain was 0.059, which is just above the threshold of 0.05, and this deletion strain was highly significant in Fig. 2B in the main text. Standard growth of the strains was confirmed in SD medium with 2% glucose (data not shown). Download FIG S2, EPS file, 0.8 MB. Copyright © 2022 Bui et al. 2022 Bui et al. https://creativecommons.org/licenses/by/4.0/ This content is distributed under the terms of the Creative Commons Attribution 4.0 International license.

### *CgTUP11* represses more genes than *CgTUP1*, but the two appear to have an overlap of target genes.

**FIG 4:**
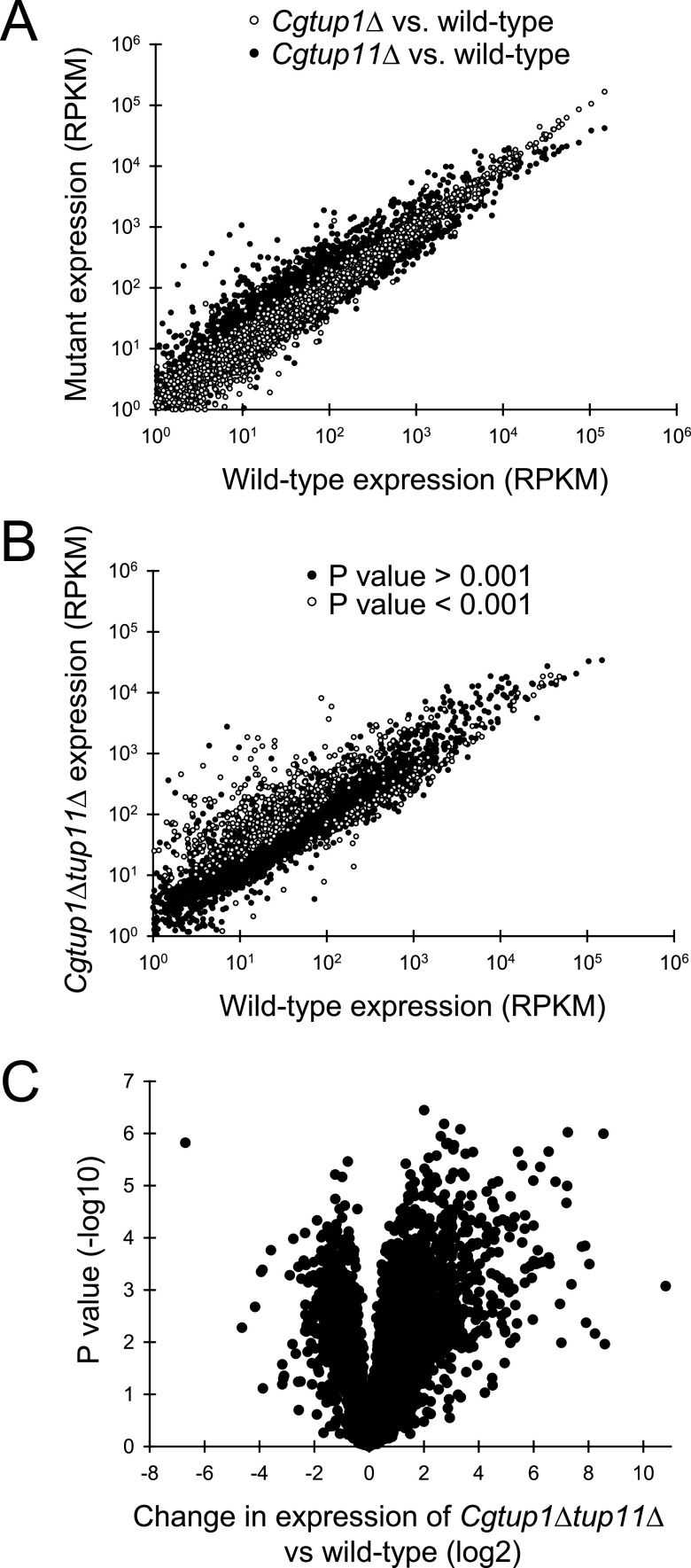
RNA-seq of *Cgtup1*Δ, *Cgtup11*Δ, and *Cgtup1*Δ *Cgtup11*Δ strains compared to the wild type. (A) Comparison of *Cgtup1*Δ RNA expression (RPKM) (unfilled circles) and *Cgtup11*Δ RNA expression (RPKM) (filled circles) relative to wild-type RPKM. Two biological replicates of the *Cgtup1*Δ and *Cgtup11*Δ strains are compared to three biological replicates of wild-type and the mean expression is indicated. Genes that were expressed at less than 2 RPKM were not graphed, as their expression level was considered too low to be accurate. (B) Comparison of *Cgtup1*Δ *Cgtup11*Δ RNA expression (RPKM) (three biological replicates) relative to the wild type (three biological replicates). (C) Volcano plot of data from panel B, where the *x* axis is the log_2_ change in expression (RPKM) of the *Cgtup1*Δ *Cgtup11*Δ strain versus the wild type, and the *y* axis is the –log_10_ of the *P* value as determined by a Student *t* test.

To understand the role that *CgTUP1* and *CgTUP11* have in transcription, we performed a series of RNA-seq (transcriptome sequencing) experiments with each individual deletion strain and the double-deletion strain, comparing them to the wild type. RNA was isolated from actively dividing cells in standard yeast extract, peptone, and dextrose (YEPD) medium. Comparison of RNA expression of the two *Cgtup1*Δ and *Cgtup11*Δ deletion strains to the wild type indicated that *CgTUP11* was a more active repressor of gene expression than *CgTUP1* (Fig. 4A). For example, there are 19 genes that increase over 20-fold in response to deletion of *CgTUP11*, and there were no genes that changed that much in response to *CgTUP1* deletion. Additionally, the deviation of the mutant expression from that of the wild type is much more apparent in the *Cgtup11*Δ/wild-type comparison relative to the *Cgtup1*Δ/wild-type comparison (Fig. 4A).

Examination of the genes that appear to be most upregulated in response to deletion indicated that there is some overlap between the targets of repression of the two paralogs. For example, *CgFBP1* and *CgHSP30* are both derepressed in each deletion (Table S1). Because the individual mutants were only analyzed in duplicate RNA-seq experiments, we also analyzed the double mutant (*Cgtup1*Δ *Cgtup11*Δ) relative to the wild type (both performed in biological triplicate), which allowed us to determine statistical significance (Fig. 4B). Measuring expression of the double mutant relative to that of the wild type demonstrated that the double mutant had a larger change in expression of more genes than each single mutant, and thus, we concluded that the double mutant has more derepression than each single mutant. Plotting the same data as a volcano plot (Fig. 4C) identified 471 genes that that are derepressed in the double mutant 2-fold with a *P* value of less than 0.001.

10.1128/mSphere.00765-21.5 TABLE S1 RNA-seq data. Download Table S1, XLSX file, 1.5 MB. Copyright © 2022 Bui et al. 2022 Bui et al. https://creativecommons.org/licenses/by/4.0/ This content is distributed under the terms of the Creative Commons Attribution 4.0 International license.

Using a more stringent value of 4-fold change and a *P* value of <0.001, there are 248 genes that were derepressed in the double mutant and 15 genes that were repressed. These data suggest that the *TUP1* paralogs are much more important for repression than for gene activation. There is no clear grouping of the genes that are responsive to Tup1 activation, other than *CgEPA15* being the most repressed in the double mutant, at 105-fold repression. A gene ontology (GO) analysis of the 248 derepressed genes indicated a slight enrichment for carbohydrate metabolic processes, glycogen metabolic processes, and polysaccharide metabolism, which are consistent with targets of *ScTUP1* and may explain the growth advantage on nonfermentable carbon sources. These data in total suggest that *CgTUP11* is the most similar in function to *ScTUP1* but that *CgTUP1* is capable of repressing some of the same genes as well as other genes to a lesser extent. One simple explanation for the phenotypes could be that *CgTUP11* is expressed at a higher level. While we cannot eliminate the possibility that there are different amounts of the two proteins, the transcript abundance of both genes was observed at a statistically identical level in the wild-type RNA-seq data (21.6 reads per kilobase per million [RPKM] for *CgTUP11* versus 20.7 RPKM for *CgTUP1*).

### RT-qPCR validates RNA-seq and indicates that targets have variability in sensitivity to *CgTUP1*, *CgTUP11*, and *CgCYC8*.

**FIG 5:**
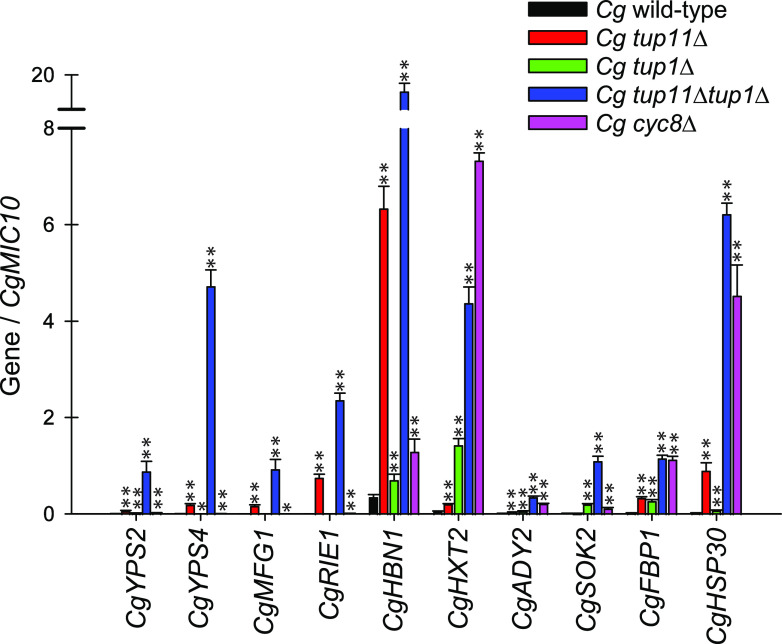
RT-qPCR of candidate C. glabrata genes in *tup*Δ and *cyc8*Δ strains. C. glabrata wild-type and mutant strains were grown in standard YEPD medium to logarithmic growth phase. RNA was harvested and reverse transcribed to cDNA, and quantitative PCR was performed using primers for candidate genes as determined by the RNA-seq data in Fig. 4. The amount of transcript was normalized to *CgMIC10*, which did not change expression with the loss of *TUP* genes. The data presented are the means and standard deviations of three biological replicates. *P* value was determined by a Student *t* test comparing each deletion strain to the wild type, with a single asterisk indicating a *P* value of <0.05 and a double asterisk indicating a *P* value of <0.01.

To confirm the targets identified in the RNA-seq data set, we harvested RNA from various strains grown in triplicate and performed reverse transcription-quantitative PCR (RT-qPCR) on candidate genes, which were some of the most derepressed genes in the double mutant in the RNA-seq data set (Fig. 5). We used the gene *CgMIC10* for normalization, as it did not change expression in response to loss of *TUP1* paralogs and was highly expressed (based on RPKM, it was in the top 5% of expressed genes). Whereas all of these genes change expression in a statistically significant manner in at least three of the mutant strains, some of the genes appear to be primarily regulated by *CgTUP11* (such as *CgYPS2*, *CgYPS4*, *CgMFG1*, *CgRIE1*, and *CgHBN1*), others are primarily regulated by *CgTUP1* (*CgHXT2* and *CgSOK2*), and others appear to be regulated by both (*CgFBP1* and *CgHSP30*). With many genes, there is a significant effect when both *TUP* homologs are deleted: i.e., the double-deletion strain is even more derepressed than individual mutants. Interestingly, all of these target genes appear to require *CgCYC8* for repression, but the strongly *CgTUP11*-dependent genes (*CgYPS2*, *CgYPS4*, *CgMFG1*, *CgRIE1*, and *CgHBN1*) appear to not mirror the *Cgtup11*Δ *Cgtup1*Δ double mutant, suggesting that maybe another corepressor is important for full repression. There are two proteins annotated as being related to *ScCYC8* in the C. glabrata genome; we deleted *CAGL0D01364g*, which is 84% identical to *ScCYC8* over the region corresponding to ∼400 aa (bit score, 812), but *CAGL0M01914g* has 21% identity over the same region (bit score, 41). It is possible other corepressors are more important for *Cg*Tup11 interactions. Importantly, the RT-qPCR analysis of genes on independently grown cultures from the RNA-seq data set validates that the genes we have identified as targets in RNA-seq experiments are likely genuine. Further mechanistic analysis of how these two Tup1 proteins interact with Cyc8, and potentially other proteins, is needed to dissect the different classes of genes regulated by the *TUP1* paralogs.

### *CgTUP11* complements the *Sctup1*Δ strain based on RNA-seq and *CgTUP1* regulates only half of the same genes as *ScTUP1*.

**FIG 6:**
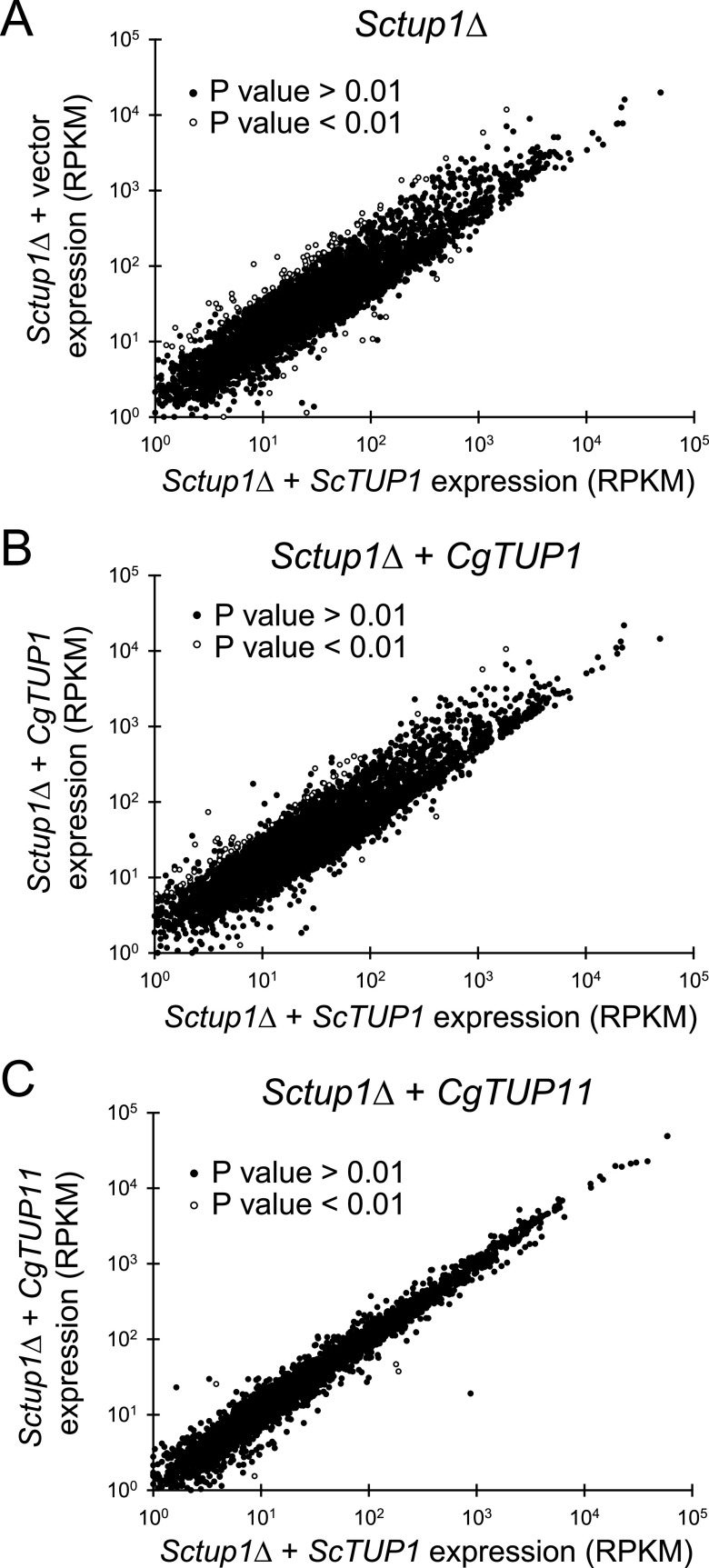
RNA-seq of *Sctup1*Δ with complementing plasmids. (A) Comparison of *Sctup1*Δ plus vector RNA expression (RPKM) and *Sctup1*Δ with *ScTUP1* RNA expression (RPKM). Three biological replicates of each strain are compared and the mean expression is plotted. Genes that were expressed at less than 2 RPKM in both strains were not graphed. (B) Comparison of *Sctup1*Δ plus *CgTUP1* RNA expression (RPKM) and *Sctup1*Δ with *ScTUP1* RNA expression (RPKM). (C) Comparison of *Sctup1*Δ plus *CgTUP11* RNA expression (RPKM) and *Sctup1*Δ with *ScTUP1* RNA expression (RPKM).

**FIG 7:**
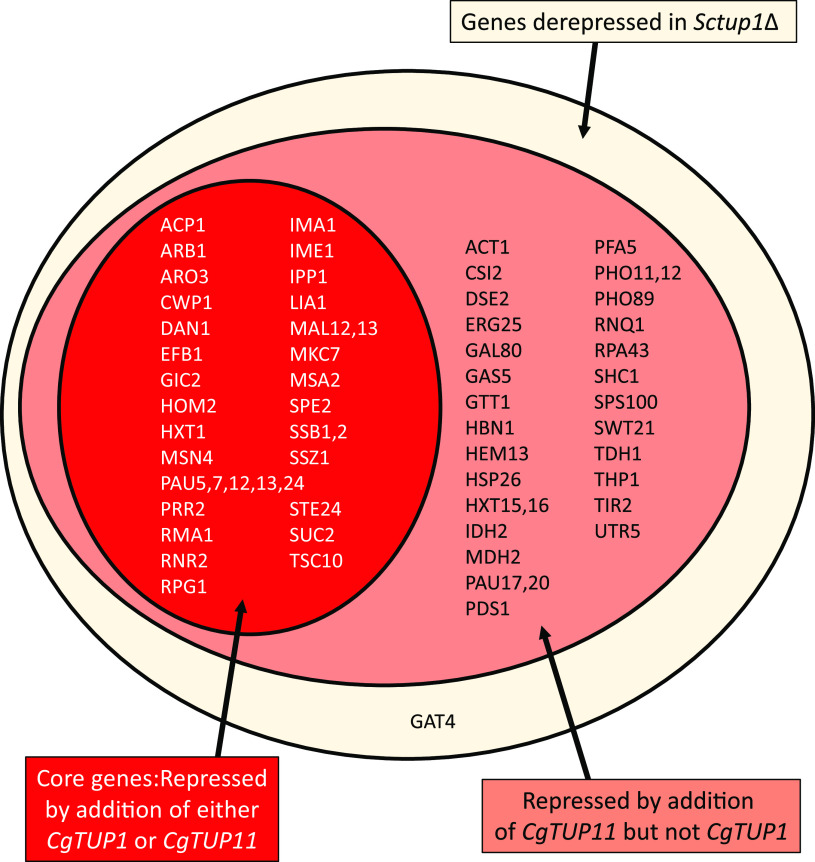
Diagram of genes identified in RNA-seq of *Sctup1*Δ strain with complementing plasmids. There are 68 three-letter-named genes in S. cerevisiae that are upregulated more than 4-fold (*P* < 0.01) in the *Sctup1*Δ strain, indicated by the yellow circle. Thirty-six of those genes are repressed by addition of either *CgTUP1* or *CgTUP11* (red), and the other 32 are only repressible by *CgTUP11* (pink).

To compare the regulation of C. glabrata genes with that of S. cerevisiae genes in response to deletion of *TUP1* homologs, we performed RNA-seq with the *Sctup1*Δ strain, where we added back different versions of *TUP1* in a plasmid. First, we examined the *Sctup1*Δ strain with an empty vector relative to a plasmid containing the wild-type version of *ScTUP1* (Fig. 6A), allowing us to identify the targets for repression by *Sc*Tup1. To confirm that our *Sctup1*Δ strain behaved like in previous studies, we compared known *Sc*Tup1 targets in the *Saccharomyces* Genome Database (SGD) with our targets and found that >60% of known targets in SGD also changed expression in a statistically significant manner in our RNA-seq data set. We identified 71 genes that were derepressed 4-fold (*P* < 0.01) in response to loss of *ScTUP1*, and the GO annotation indicated that these genes are enriched for fungal cell wall and external encapsulating structure organization and sucrose metabolic processes, which is not surprising given the known phenotypes of the *Sctup1*Δ strain. We also identified 18 genes that increase expression 4-fold (*P* < 0.01) in the *Sctup1*Δ strain, and these genes are weakly enriched for mitochondrial electron transport. We then compared the *Sctup1*Δ strain with *ScTUP1* on a plasmid with the strain containing *CgTUP1* on a plasmid (Fig. 6B). In this case, there were still 52 genes derepressed 4-fold (*P* < 0.01), and 63% of those derepressed genes were represented in the 71 genes identified in the *Sctup1*Δ alone. These data suggest that *CgTUP1* is capable of complementing only some of the defects of the *Sctup1*Δ strain. However, when we added back *CgTUP11* to the *Sctup1*Δ strain (Fig. 6C), the strain behaved very similarly to the wild type (*Sctup1*Δ plus *ScTUP1*), with only 2 of the 71 *ScTUP1*-dependent genes still being upregulated 4-fold. Specifically examining the genes that have three-letter names associated with them (68 of the 71 genes) that are upregulated in the *Sctup1*Δ strain, all but *ScGAT4* are repressed by the addition of *CgTUP11* (Fig. 7). Addition of *CgTUP1*, however, represses only 53% of the *ScTUP1* targets. Overall, the data are consistent with *CgTUP11* complementation of the flocculation phenotype in the S. cerevisiae *tup1* mutant (Fig. 3), indicating that *CgTUP11* can functionally replace *ScTUP1*, and the RNA-seq data indicate that *CgTUP1* can partially substitute for *ScTUP1.* Interestingly, there are new targets identified in the *Sctup1*Δ plus *CgTUP1* strain that are not repressed, suggesting that *CgTUP1* has a different specificity from *ScTUP1* in S. cerevisiae (Fig. S3).

10.1128/mSphere.00765-21.3 FIG S3 Diagram of genes identified in RNA-seq of the *Sctup1*Δ strain with complementing plasmids. There are 68 three-letter-named genes in S. cerevisiae that are upregulated more than 4-fold (*P* < 0.01) in the *Sctup1*Δ strain, indicated by the circle on the left (yellow and orange). Thirty-six of those genes are repressed by addition of either *CgTUP1* or *CgTUP11* (yellow), and the other 32 are only repressible by *CgTUP11* (orange). Genes in the red circle are new targets identified in the *Sctup1*Δ plus *CgTUP1* strain that are not repressed, suggesting that *CgTUP1* has a different specificity from *ScTUP1* in S. cerevisiae. Finally, only two genes are derepressed in the *Sctup1*Δ plus *CgTUP11* strain, indicating that *CgTUP11* functionally replaces *ScTUP1*; *GAT4* is not a target of either *CgTUP11* or *CgTUP1*, and *DIT1* is a new target of repression by both *CgTUP11* and *CgTUP1*. Download FIG S3, EPS file, 1.9 MB. Copyright © 2022 Bui et al. 2022 Bui et al. https://creativecommons.org/licenses/by/4.0/ This content is distributed under the terms of the Creative Commons Attribution 4.0 International license.

To determine whether *in vivo* the *TUP1* homologs regulate similar genes, we examined the top 100 genes from our RNA-seq data sets that were regulated by *ScTUP1* and by the two C. glabrata homologs (Fig. S4). We then assessed whether there was a clear homolog in the other species and determined whether that homolog was also upregulated in a significant manner (*P* value < 0.05 in a Student *t* test). It is worth mentioning that for many gene families, including those encoding the yapsins (*YPS*), adhesins (*EPA*), and hexose transporters (*HXT*), it is difficult to discriminate true orthologs, and so we collapsed gene families into one representative and assessed whether they were similarly regulated. We determined that 37% of the S. cerevisiae genes that are upregulated (with a *P* value <0.05) in the *Sctup1*Δ strain are also upregulated in the *Cgtup1*Δ *tup11*Δ strain. Conversely, 43% of the upregulated *Cgtup1*Δ *tup11*Δ genes were also upregulated in the *Sctup1*Δ strain. We conclude from these analyses that *ScTUP1*, *CgTUP11*, and *CgTUP1* do target some of the same genes, but likely because of speciation, and divergence of the two paralogs in C. glabrata, there are differences in the specific targets in each species.

10.1128/mSphere.00765-21.4 FIG S4 While *ScTUP1*, *CgTUP1*, and *CgTUP11* do target some of the same genes, there are differences in the specific targets. (A) The top 100 genes regulated by *ScTUP1*, as determined by RNA-seq, were examined in the C. glabrata genome to determine if there was an ortholog and if that ortholog was regulated by *CgTUP1* and *CgTUP11* in a significant manner (*P* value < 0.05 in a Student *t* test). (B) Conversely, the top 100 genes regulated by *CgTUP1* and *CgTUP11*, as determined by RNA-seq of the *Cgtup1*Δ *tup11*Δ strain, were examined in the S. cerevisiae genome to determine if there was an ortholog and if that ortholog was regulated by *ScTUP1*. Download FIG S4, EPS file, 2.0 MB. Copyright © 2022 Bui et al. 2022 Bui et al. https://creativecommons.org/licenses/by/4.0/ This content is distributed under the terms of the Creative Commons Attribution 4.0 International license.

## DISCUSSION

We have identified a *TUP1* paralog (*CgTUP11*, or *CAGL0E00561g*) in C. glabrata that is equally as important as, if not more important than, the known *CgTUP1* (*CAGL0C03608g*) gene in standard growth medium. After the WGD and dramatic loss of most paralogs, the retention of two *TUP1* paralogs in post-WGD species is likely important. It is possible that this allows for more specialization or exploitation of different niches, but it is noteworthy that the *Saccharomyces* species complex appears to have only one homolog. It is possible that S. cerevisiae has simplified its ability to repress genes: i.e., there can be an ON/OFF switch for stress or no stress. Conversely, C. glabrata and related species may use the two paralogs to tailor multiple repressive regimes for varied stress conditions. Although we do not know the independent and overlapping roles of the duplicate *TUP1* genes in C. glabrata, lessons from Schizosaccharomyces pombe, in which there are two homologs, may be informative. In S. pombe, Tup11 and Tup12 have both functionally redundant and distinct functions, but Tup12 appears to have more specific repression activity than Tup11 (27).

Our data implicate C. glabrata *TUP1* and *TUP11* in pathogenicity. For example, previous work has suggested that *Cg*Tup1 is recruited by Yap7 (part of the Yeast AP1 family) to repress *YHB1*; *YHB1* encodes a gene for flavohemoglobin, which detoxifies nitric oxide (26). We were unable to see a significant differential effect of nitric oxide stress on the *Cgtup1*Δ *tup11*Δ strain relative to the wild type (data not shown), but it raises the possibility that the C. glabrata *TUP1* paralogs might be important for survival in mammalian cells. Additionally, yapsin (aspartyl proteases) genes are important for C. glabrata survival in macrophages and cell wall structure and thereby have direct involvement in the species’ pathogenicity (28). Examination of the *YPS* gene family in the RNA-seq data indicated that *YPS2*, *YPS4*, *YPS6*, *YPS8*, *YPS9*, and *YPS10* have increased expression in the absence of both *TUP1* homologs. Given that 6 of the 11 *YPS* genes have increased expression (and *YPS4* has a 152-fold increase in expression in the double mutant), a better understanding of how the C. glabrata Tup1 homologs contribute to pathogenicity is needed.

Finally, it is surprising that we observed few clear, robust phenotypes in the *Cgtup1*Δ *tup11*Δ or the *Cgcyc8*Δ strain, especially in light of many genes being repressed by these proteins. The simplest explanation would be that the genes were not actually deleted, but the RNA-seq data are strongly indicative of the appropriate gene deletion. *Sctup1*Δ strains grow slower than the wild type and can access alternative carbon sources. It is easy to think of *ScTUP1* as repressing many stress genes, and loss of *ScTUP1* results in many stress genes being upregulated, causing slow growth. In C. glabrata, a survey of many stress conditions did not identify clear phenotypes. Additionally, the deletion of *CgCYC8* suggested that there are genes that are strongly dependent on the *TUP1* paralogs but not as dependent on *CYC8*, such as *CgYPS4* or *CgHBN1*. This suggests that the well-defined complexes described for S. cerevisiae are not so canonical in other species, and possibly other proteins are required for repression in Tup1-containing complexes (17, 19). The altered lack of repression of genes by *CgTUP1* in the *Sctup1*Δ strain (both partial repression of *ScTUP1* targets and additional genes not thought to be regulated by *ScTUP1*) supports the notion that there are likely multiple complexes that target different genes. Additionally, supporting the argument of additional complexity in C. glabrata is the observation that very few genes are downregulated in response to deletion of the *TUP1* homologs in C. glabrata. This is in contrast to the case with S. cerevisiae, for which there are demonstrated activation roles for the Tup1-Cyc8 complex (29, 30). Further dissection of the Tup1 and Tup11 complexes is warranted to understand the differential roles of these complexes in gene repression in post-WGD species.

## MATERIALS AND METHODS

### Strains and plasmids.

Strains used in this study are listed in Table S2. Genes were deleted in the C. glabrata wild-type strain using antibiotic resistance markers, *KANMX6* and *NATMX6*, which replaced open reading frames via homologous recombination (primers listed in Table S3). Deletions were verified using gain of the selectable marker as well as PCR to confirm loss of the open reading frame and positivity for flanking PCR regions.

10.1128/mSphere.00765-21.6 TABLE S2 Strains used in this study. Download Table S2, PDF file, 0.5 MB. Copyright © 2022 Bui et al. 2022 Bui et al. https://creativecommons.org/licenses/by/4.0/ This content is distributed under the terms of the Creative Commons Attribution 4.0 International license.

10.1128/mSphere.00765-21.7 TABLE S3 Primers used in this study. Download Table S3, PDF file, 0.5 MB. Copyright © 2022 Bui et al. 2022 Bui et al. https://creativecommons.org/licenses/by/4.0/ This content is distributed under the terms of the Creative Commons Attribution 4.0 International license.

*URA3*^+^ plasmids (pRS316) containing *TUP* genes were used for cross complementation experiments with an *Sctup1*Δ strain. Empty pRS316 plasmid (vector) was used as a negative control, and pRS316 containing wild-type *ScTUP1* was used as a positive control. Plasmids containing *CgTUP1* and *CgTUP11* were also transformed into the *Sctup1*Δ strain. The primers for construction of the plasmids by gap repair are listed in Table S3 (31).

*HIS3*^+^ plasmids (pRS313) containing *TUP* genes were used for cross complementation experiments with *Cgtup1*Δ and *Cgtup11*Δ strains. The primers for construction of the plasmids by gap repair are listed in Table S3.

### Phenotypic assays.

To investigate visible phenotypic differences between deletion strains and wild-type, strains were grown on various plate and liquid medium conditions. The experimental plate conditions were 1 M potassium chloride, 0.25 M calcium chloride, 110 μM inositol, 0.5 μg/mL of ketoconazole, and high temperature (37°C) in yeast extract, peptone, dextrose (YEPD) standard medium. Each plate was divided into six subsections for S. cerevisiae wild-type and *Sctup1*Δ and C. glabrata wild-type, *Cgtup1*Δ, *Cgtup11*Δ, and *Cgtup1*Δ *tup11*Δ strains. From 5 mL of YEPD liquid cultures grown overnight, colonies were streaked for single colonies. Growth was assessed after 24 h at 30°C for all conditions except high temperature, which was grown at 37°C for 24 h.

To assess phenotypes in liquid media, the S. cerevisiae wild-type and *Sctup1*Δ strains and C. glabrata wild-type, *Cgtup1*Δ, *Cgtup11*Δ, *Cgtup1*Δ *tup11*Δ, and *Cgcyc8*Δ strains were grown under various conditions. In standard yeast extract and peptone (YEP) medium, different carbon sources replaced the 2% glucose: 1% ethanol and 1% glycerol together, 0.2% glucose, 2% ethanol, and 2% acetic acid. In standard yeast extract, peptone, and dextrose (YEPD) medium, various compounds were added: 9 mM 2-deoxyglucose, 1 mM FeCl_3_, 2.8 mM Congo red, 1 M potassium chloride (KCl), 10 mM Tris (pH 7.9), and 0.5 μg/mL of ketoconazole. Strains were grown in standard YEPD overnight, and then cells were harvested and washed three times with water. Cultures were inoculated at an optical density at 600 nm (OD_600_) of 0.05 in triplicate. After 24 to 48 h of growth at 30°C, the optical density of each culture was measured to determine differences in growth. A subset of these conditions was retested using synthetic defined (SD) medium instead of standard YEP (Fig. 2B and Fig. S1). For the data presented in Fig. S1, six biological replicates of each strain were inoculated at an OD_600_ of 0.01 in 96-well plates rather than in culture tubes.

To assess the phenotype for growth in liquid medium containing sorbitol instead of glucose, S. cerevisiae wild-type and *Sctup1*Δ strains and C. glabrata wild-type, *Cgtup1*Δ, *Cgtup11*Δ, *Cgtup1*Δ *tup11*Δ, and *Cgcyc8*Δ strains were precultured in YEPD overnight and then in YEP with 3% glycerol overnight. The cells were washed three times with water and inoculated into SD medium with either 2% sorbitol or 2% glucose at an OD_600_ of 0.05 in triplicate. After 24 to 48 h of growth at 30°C, the optical density of each culture was measured. It is worth noting that we were unable to observe a difference in growth in YEP plus 2% sorbitol, whereas the effect was dramatic in SD, suggesting that components of the media can influence the observation of phenotypes.

To measure flocculation rates, the change in OD_600_ over time was measured after vigorous vortexing. The *Sctup1*Δ strain was transformed with *URA3^+^* plasmids containing *ScTUP1*, *CgTUP1*, *CgTUP11*, or no gene (vector). These strains were grown in SD medium without uracil for ∼20 h at 30°C. Each sample was vortexed for 30 s, and the OD_600_ was recorded every 15 s for 1 min.

### RT-qPCR.

C. glabrata wild-type, *Cgtup1*Δ, *Cgtup11*Δ, *Cgtup1*Δ *tup11*Δ, and *Cgcyc8*Δ strains were grown in triplicate in YEPD for ∼20 h, then inoculated at a low density in fresh YEPD medium, and grown for 5 h at 30°C to logarithmic growth phase. RNA was extracted using the Zymo Research Corp. Direct-zol RNA MiniPrep Plus kit and reverse transcribed to cDNA using the Bio-Rad iScript cDNA synthesis kit. Quantitative PCR was performed with a CFX qPCR machine (Bio-Rad) using Bio-Rad SsoAdvanced Universal SYBR green Supermix in a 25-μL reaction mixture. The amount of transcript for each gene was normalized to *CgMIC10*, which has consistent expression across the various strains, unlike the more common normalization control *CgACT1*. Each gene was also amplified using 10-fold genomic DNA dilutions as an amplification control. Based on RNA-seq data, we targeted genes that exhibited elevated transcription levels in *tup1* mutants. Genes and primer sequences can be found in Table S3.

### RNA-seq.

RNA sequencing was performed on two sets of strains, one set to look at expression in C. glabrata and one set to look at expression in S. cerevisiae. C. glabrata wild-type, *Cgtup1*Δ, *Cgtup11*Δ, and *Cgtup1*Δ *tup11*Δ strains were grown in YEPD standard medium for 6 h at 30°C. Wild-type and *Cgtup1*Δ *tup11*Δ strains were grown in triplicate, but *Cgtup1*Δ and *Cgtup11*Δ strains were only grown in duplicate. The *Sctup1*Δ strain was transformed with *URA3^+^* plasmids containing *ScTUP1*, *CgTUP1*, *CgTUP11*, or no gene (vector). These strains were grown to logarithmic growth phase in SD medium without uracil for 6 h at 30°C in triplicate.

RNA was purified using the Zymo Research Corp. Direct-zol RNA MiniPrep Plus kit, and the concentration of RNA was determined using a Qubit 3.0 fluorometer and the Qubit RNA HS assay kit. The RNA library was prepared using the NEBNext Ultra II RNA library prep kit for Illumina protocol from New England BioLabs, Inc. Samples were diluted in 0.1× Tris-EDTA (TE) to 4 nM and the Illumina NextGen MiSeq sequencer was used to sequence the samples, generating FASTQ files for each sample that were >1 million reads. Single-end reads were paired, trimmed, and aligned to the reference C. glabrata or S. cerevisiae genomes using Geneious (using default settings), and RPKM for each gene was exported for analysis. FASTQ files are deposited in the NCBI SRA database (accession number PRJNA782995).

To analyze the RNA sequencing data, genes were sorted by ratio of expression between the mutant and wild-type strains. In the case of C. glabrata, the *Cgtup1*Δ *tup11*Δ strain was used as the mutant for obtaining ratios. For S. cerevisiae, *Sctup1*Δ with the empty vector was compared to *Sctup1*Δ with *ScTUP1*. A two-tailed Student *t* test was used to compare expression levels between mutant and wild type. The genes were then sorted by the *P* values acquired from the *t* test. For the top 100 genes that were highly expressed (*P* < 0.05), expression was compared to the expression of the corresponding homolog in the other species and it was noted whether the difference in expression between strains was significant.

### Data availability.

RNA-seq data files are available as FASTQ files in the NCBI SRA database (accession no. PRJNA782995). Experiments with mutants and the wild type and their biological replicates are available as accession numbers SAMN23411010 through SAMN23411031. Analysis of FASTQ files is available in Table S1.
