# Supplementary material for: Tup1 Paralog CgTUP11 Is a Stronger Repressor of Transcription than CgTUP1 in Candida glabrata
Source: mSphere. 2022 Mar 28;7(2):e00765-21. doi: 10.1128/msphere.00765-21 (PMC9044973; doi:10.1128/msphere.00765-21)
Supplement: TABLE S2 [file msphere.00765-21-st002.pdf]

Table S2. Strains used in this study.

Name	Genotype
DC3	*S. cerevisiae* wild-type K699 *ade2-1 trp1-1 can1-100 leu2-3,112 his3-11,15 ura3*
DC96	*S. cerevisiae* tup1::LEU2 ade2-1 trp1-1 can 1-100 leu2-3,112 his3-11,15 ura3 GAL+ (from K699)
DG5	*C. glabrata* wild-type (*his3-*) (BG99)
DG176	*C. glabrata* wild-type *(his3- ura3ΔKANMX6)*
DG371	*Cgtup1ΔNATMX6* (in DG5)
DG370	*Cgtup11ΔKANMX6* (in DG5)
DG372	*Cgtup11ΔKANMX6 tup1ΔNATMX6* (in DG370)
DG459	*Cgcyc8ΔNATMX6* (in DG176)
DG460	*Cgtup11ΔNATMX6* (in DG176)
DG461	*Cgtup1ΔNATMX6* (in DG176)
DB14	pRS316 (*URA3+*)
DB511	*ScTUP1* -pRS316 (*URA3+*)
DB509	*CgTUP1* -pRS316 (*URA3+*)
DB510	*CgTUP11* -pRS316 (*URA3+*)
DB2	pRS313 (*HIS3+*)
DB547	*ScTUP1* -pRS313 (*HIS3+*)
DB545	*CgTUP1* -pRS313 (*HIS3+*)
DB546	*CgTUP11* -pRS313 (*HIS3+*)
