# Supplementary material for: Tup1 Paralog CgTUP11 Is a Stronger Repressor of Transcription than CgTUP1 in Candida glabrata
Source: mSphere. 2022 Mar 28;7(2):e00765-21. doi: 10.1128/msphere.00765-21 (PMC9044973; doi:10.1128/msphere.00765-21)
Supplement: TABLE S3 [file msphere.00765-21-st003.pdf]

Table S3. Primers used in this study.

		**Primers to delete genes**
Cgtup1ΔNATMX6	1832	ATGGCTAAGGAGAAGGATGTTATAGGCGGGCATGCTGCGAcggatccccgggttaattaa
	1833	TATCATCATGATATTAGCATTATCATGGTTATGACACGAAGAATTCGAGCTCGTTTAAAC
	1852	GTGTAGTAGTTGGGATGGCCTGGATGGGGGGCAAGGCGCTATGGCTAAGGAGAAGGATG
	1853	CATAAGAAAAGAAAGAGAAACAAAGTAGATGATATAGCTATCATCATGATATTAGCATT
Cgtup11ΔKANMX6	2292	ATGACCGCTAACGCTTCACTAGGTAAGCTGAATGATCTCCCGGATCCCCGGGTTAATTAA
	2293	TGTTCGGTTTATGAAATTGGTTGGATCTTTCCATTTGAATGAATTCGAGCTCGTTTAAAC
	2320	TTTTGAGAATACTGATAAACTTGTTTGAACTTAGAGAAACCAATGACCGCTAACGCTTCA
	2321	AATAATAATAAACAACAAATATTAAAATATTATTGTCTTCTGTTCGGTTTATGAAATTGG
Cgcyc8ΔNATMX6	3087	CTCTCTCTTTGCGAAAACTAGATAACCACGCTTAAGAATA**CGGATCCCCGGGTTAATTAA**
	3088	AAATAAATGATTACATCATGATTGTGAATCTGCGGATCTA**GAATTCGAGCTCGTTTAAAC**
		**Primers to put genes into plasmids**
ScTUP1 -pRS316 or -pRS313	3149	ggagctccaccgcggtggcggccgctctagaactagtAAGTCTGCGGAATCGATCTG
	3150	cggtatcgataagcttgatatcgaattcctgcagcccgggATCAAAGAATAATGAACCGC
CgTUP1 -pRS316 or -pRS313	3081	ggagctccaccgcggtggcggccgctctagaactagtGCTCCTTGAGGTGCTTGATG
	3082	cggtatcgataagcttgatatcgaattcctgcagcccgggCAATTGTTGAATAATACATC
CgTUP11 -pRS316 or -pRS313	3084	ggagctccaccgcggtggcggccgctctagaactagtGTCAAAAAGATAAACAGTTG
	3085	cggtatcgataagcttgatatcgaattcctgcagcccgggTTGACCAACAGATTAGGTCC
		**Primers for qPCR**
CgYPS2 (CAGL0E01419g)	2736	TGATCTTCATAGTCTAGTTG
	2737	CAGTAGGATTCCTGGCTCAC
CgYPS4 (CAGL0E01749g)	2740	TTACCAGCAAAGGTTGCAGCAC
	2741	CAATGCTGCAAATTCAATGTC
CgMFG1 (CAGL0C05467g)	3182	GATGAATGATTATAGTAGCC
	3183	AGAACTATCGACATATATCG
CgRIE1 (CAGL0F08217g)	3178	AGAGAAACCATAGCTCAGCC
	3179	GTTGGCGTTTGGTAGTATCC
CgHBN1 (CAGL0B00990g)	3308	CTTCTTCACTGATGACAAGACC
	3309	GATCTTGGATGGCAAAGCAGCC
CgHXT2 (CAGL0I00286)	3306	TCTGAAACTTATCCATTGCGTG
	3307	GATGGTGGAACCCAGTTTGGAG
CgADY2 (CAGL0L07766g)	3207	ATCAATTAAGAAGTGCACTAGG
	3208	CAGTGGTATCTTCTTGACAATG
CgSOK2 (CAGL0M07634g)	3209	GATGTAGTGAAGGGCGGTCC
	3210	ATGAGTTGTTGTGACTGGAC
CgFBP1 (CAGL0H04939g)	3184	TGGGATGAATTGATAGTCTC
	3185	TGTGCTTTGGAACCAGATCC
CgHSP30 (CAGL0K07337g)	3302	TCGTCGCTTTGCTGGTTGGTGC
	3303	CACAGATTCAGGGTCTTCAACC
CgMIC10 (CAGL0B00396g)	3248	GACTGTTGACTACACACCTG
	3249	CACTGATCTCAAACCTGCAG
